# Amyand's Hernia Complicated by Omental Infarct Presenting as Acute Scrotum: Report of a Case and Review of the Literature

**DOI:** 10.1155/2015/741353

**Published:** 2015-02-16

**Authors:** Reza Khorramirouz, Amin Bagheri, Alireza Aalam Sahebpour, Abdol-Mohammad Kajbafzadeh

**Affiliations:** Department of Pediatric Urology, Children's Hospital Medical Center, Pediatric Center of Excellence, Tehran University of Medical Sciences, Tehran, Iran

## Abstract

Inguinal hernia with acute appendicitis known as Amyand's hernia is uncommon. It may clinically manifest as acute scrotum, inguinal lymphadenitis, or strangulated hernia. The presentation of Amyand's hernia with acute scrotum has been rarely described. Also, the manifestation of infarcted omentum in the inguinal hernia has been described in one case previously. However, the coexistence of perforated appendix with infarcted omentum in the hernia sac which manifests acute scrotum has not been described previously. Herein, we described a case of a 5-year-old boy, admitted with right tense, painful, and erythematous scrotum in the emergency room. The diagnosis of herniated appendicitis was performed preoperatively by ultrasound. Moreover, the ischemic omentum was confirmed during surgery.

## 1. Introduction

Amyand's hernia was first described in 1736 as a vermiform appendix in the hernia sac with or without inflammation. It may clinically manifest acute scrotum, inguinal lymphadenitis, or strangulated hernia. Acute scrotum is one of the common complaints in pediatric patients referring to the emergency room with differential diagnosis such as torsion of testis or epididymis appendix, epididymoorchitis, incarcerated hernia, scrotal skin infection, and Amyand's hernia [1–5]. The distinction between strangulated hernia and Amyand's hernia is mostly confirmed during the operation. However, only a few studies refer to the preoperative diagnosis of Amyand's hernia [1, 2]. The presentation of Amyand's hernia with acute scrotum has been rarely described and only 3 cases have been reported [1, 2]. Moreover, the incarcerated inguinal hernia with omental infarct that mimics acute scrotum is extremely rare [3]. To the best of our knowledge, the sliding hernia with perforated inflamed appendix complicated by segmental omental infarct with manifestation of acute scrotum has not been described previously. Herein, we present a unique case of inguinal hernia containing perforated appendicitis with concomitant omental infarct manifest as acute scrotum.

## 2. Case Report

**Figure 1:**
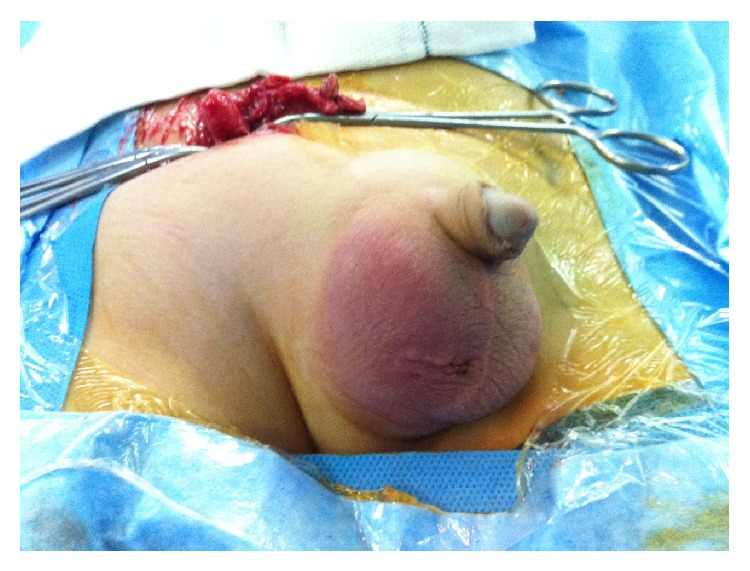
Right erythematous and testicular swelling mimicking acute scrotum.

**Figure 2:**
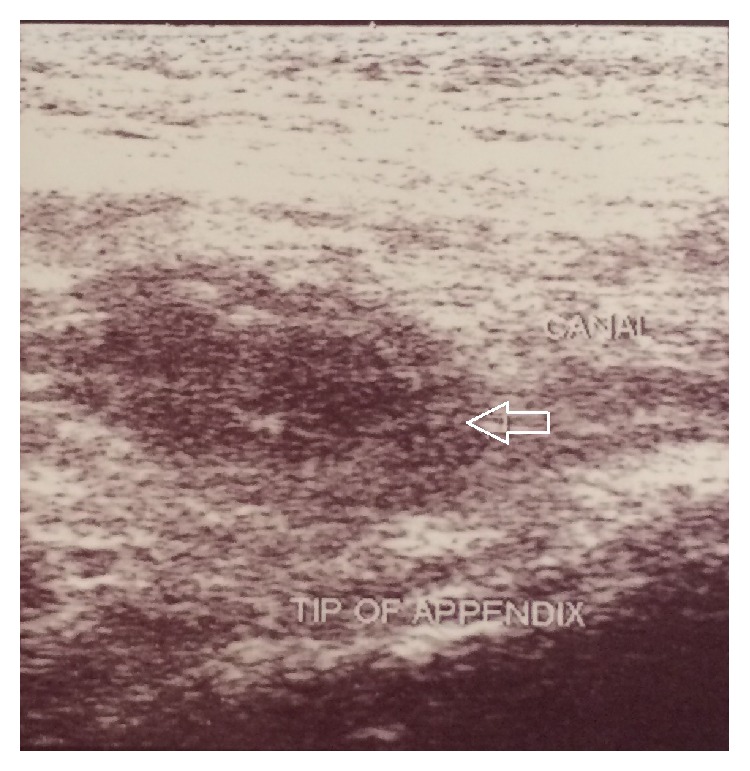
Ultrasound study shows right inguinal canal with the tip of appendix herniated into the canal (white arrow).

**Figure 3:**
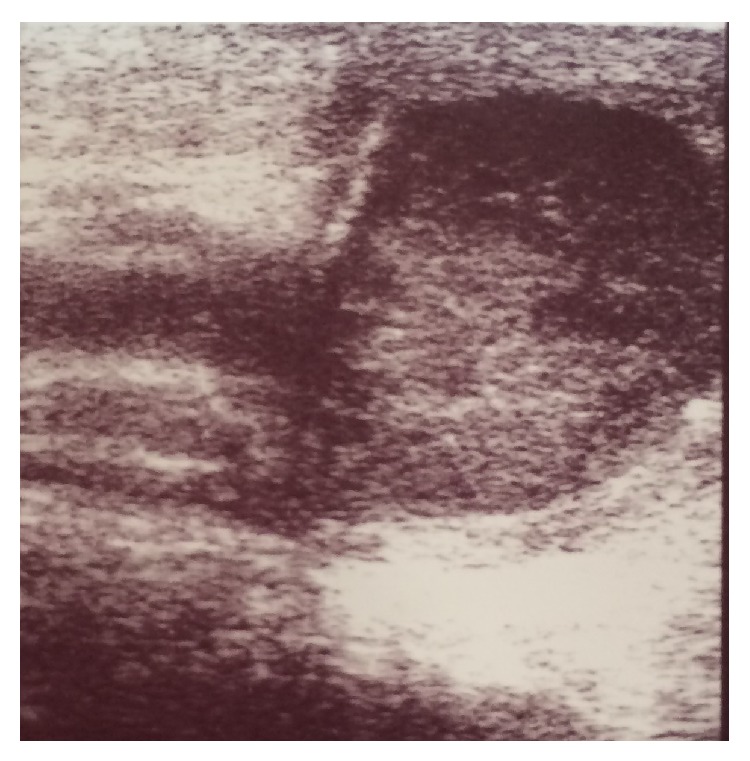
Ultrasound study shows the right testis with edematous appearance suggesting the presence of pyocele. This could be due to perforated appendicitis.

**Figure 4:**
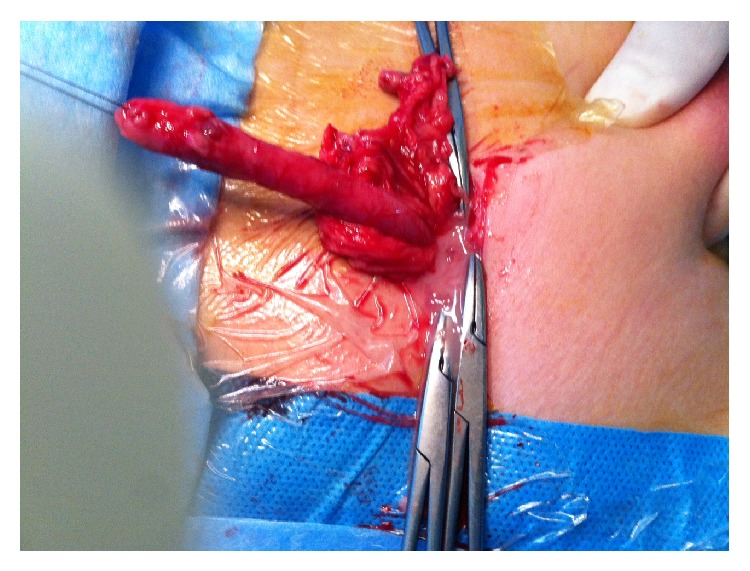
The herniated inflamed appendix was extracted by the inguinal canal.

A 5-year-old boy presented with right lower abdominal pain followed by scrotal pain. On examination, the right scrotum was firm, erythematous, and tender (Figure 1). The patient had low-grade fever and polymorphonuclear predominant white blood cell count greater than 11,000 per mm^3^ (11 × 10^9^ per L). He also had history of hydrocele and testicular pain that underwent right scrotal orchiopexy 5 months ago at district hospital. Preoperative ultrasound revealed the swollen right inguinal canal (diameter = 11 mm) accompanied by herniation of the tip of appendix into the canal. In addition, the increased wall thickness and periappendicular fat echogenicity are suggestive of gangrene and scrotal abscess (Figures 2 and 3). Although both testes had normal appearance, the right testis was edematous due to perforated appendicitis and scrotal abscess. The patient was taken to the operating room with diagnosis of acute scrotum secondary to perforated incarcerated appendicitis and scrotal abscess. Under general anesthesia through a classic incision of right inguinal hernia, hernia sac appeared swollen and erythematous. The significant part of the omentum with herniation into the sac was extracted and the ischemic part was resected subsequently. The inflamed and necrotic appendix was extracted from the herniated sac gently and released by mesoappendix (Figure 4). The cecum and appendix base was explored with normal appearance, and then the appendix was cauterized at its base. Suppurative secretion was suctioned from distal hernia sac and scrotal cavity with no evidence of intra-abdominal pus collection. The spermatic cord was intact with inflammation. Despite acute appendicitis in the hernia sac, herniorrhaphy in addition to appendectomy was performed. The patient was discharged home on the third postoperative day with good general condition.

## 3. Discussion

Amyand's hernia is a rare entity that occurs in about 0.1% of all acute appendicitis [4]. Regarding the pathophysiology of Amyand's hernia, bacterial overgrowth is a result of poor blood supply as a subsequent of either incarceration due to swelling or following the appendix entrance to the sac [5, 6]. The rare manifestation of Amyand's hernia may be acute scrotum. Testicular torsion, epididymoorchitis, incarcerated hernia, and inguinal lymphadenitis are the most common differential diagnoses of patients with Amyand's hernia present as acute scrotum. The first impression of acute scrotum in our patient was testicular torsion due to a previous history of this condition and no clinical signs of incarcerated hernia. Distinguishing between Amyand's hernia and other incarcerated hernia may be ascertained preoperatively by ultrasound. Under these circumstances preoperative ultrasound or CT scan may aid to definitive diagnosis as rarely reported previously. In present patient, preoperative ultrasound confirmed inflamed appendix within the hernia sac. Although this condition is usually diagnosed intraoperatively, only a few cases described the preoperative diagnosis of herniated appendix in the sac [2].

Moreover, this patient had concomitant infarcted omental hernia by presentation of acute scrotum as previously reported by Patel et al. [3]. Strangulated omental hernia is a rare cause of acute scrotum in the children. Omentum is a rare content of inguinal hernia in pediatrics and infarct is very exceptional [3].

Presence of acute perforated appendicitis with omental infarct in the hernia sac with suppuration determined the type of surgery and hernia repair. According to previous studies, the appendectomy for the hernia with normal appendix finding is controversial. In such cases, some authors are not favored with prophylactic appendectomy due to increased superficial and deep infection which leads to higher morbidity and recurrence, respectively [7, 8]. However, the affinity for appendectomy in pediatrics or young adult with normal herniated appendix is more than elderly because of predisposition to future acute appendicitis. But the existence of inflamed appendix in the hernia sac needs appendectomy and also the method of hernia repair is done by Bassini or Shouldice techniques, without mesh because of high risk of infection in such cases.

According to Losanoff and Basson classification of Amyand's hernia, the present case is categorized in type 2 with acute appendicitis within inguinal hernia and no abdominal sepsis that was subjected to appendectomy and primary endogenous hernia repair without mesh [9, 10].

## 4. Conclusion

The presence of acute scrotum in pediatric settings may be related to nontesticular origin such as specific type of hernia. Amyand's hernia and omental infarct may manifest clinically with acute scrotum separately in the way of perforation or incarceration as in the present case with both conditions.

## References

[1] Junaid J., Fawad A. (2012). A normal appendix in a painful sliding hernia—an unusual case. *Journal of the Pakistan Medical Association*.

[2] Çelik A., Ergün O., Özbek S. S., Dökümcü Z., Balık E. (2003). Sliding appendiceal inguinal hernia: preoperative sonographic diagnosis. *Journal of Clinical Ultrasound*.

[3] Patel R. V., Dawrant M., Scott V., Fisher R. (2014). Omental infarct in a hernia: an unusual cause of paediatric acute scrotum. *BMJ Case Reports*.

[4] Pellegrino J. M., Feldman S. D. (1992). Case report: acute appendicitis in an inguinal hernia. *New Jersey Medicine*.

[5] Weber R., Hunt Z., Kral J. (1999). Amyand’s hernia: etiologic and therapeutic implications of two complications. *Surgical Rounds*.

[6] Abu-Dalu J., Urca I. (1972). Incarcerated inguinal hernia with a perforated appendix and periappendicular abscess: report of a case. *Diseases of the Colon and Rectum*.

[7] Sharma H., Gupta A., Shekhawat N. S., Memon B., Memon M. A. (2007). Amyand's hernia: a report of 18 consecutive patients over a 15-year period. *Hernia*.

[8] D'Alia C., Lo Schiavo M. G., Tonante A. (2003). Amyand's hernia: case report and review of the literature. *Hernia*.

[9] Losanoff J. E., Basson M. D. (2007). Amyand hernia: what lies beneath—a proposed classification scheme to determine management. *The American Surgeon*.

[10] Losanoff J. E., Basson M. D. (2008). Amyand hernia: a classification to improve management. *Hernia*.

